# Smart Sanitation—Biosensors as a Public Health Tool in Sanitation Infrastructure

**DOI:** 10.3390/ijerph17145146

**Published:** 2020-07-16

**Authors:** Emma Rary, Sarah M. Anderson, Brandon D. Philbrick, Tanvi Suresh, Jasmine Burton

**Affiliations:** 1 Rollins School of Public Health, Emory University, Atlanta, GA 30322, USA; emmarary@gmail.com (E.R.); sarah.margaret.anderson@gmail.com (S.M.A.); 2 Wish for WASH Thinks, Inc, Atlanta, GA 30338, USA; brandon.philbrick@emory.edu (B.D.P.); tanvi.suresh96@gmail.com (T.S.); 3 Emory University School of Medicine, Atlanta, GA 30322, USA

**Keywords:** sanitation, toilet, sewage, wastewater-based epidemiology, global health, health sensor, biosensor, biological sensor

## Abstract

The health of individuals and communities is more interconnected than ever, and emergent technologies have the potential to improve public health monitoring at both the community and individual level. A systematic literature review of peer-reviewed and gray literature from 2000-present was conducted on the use of biosensors in sanitation infrastructure (such as toilets, sewage pipes and septic tanks) to assess individual and population health. 21 relevant papers were identified using PubMed, Embase, Global Health, CDC Stacks and NexisUni databases and a reflexive thematic analysis was conducted. Biosensors are being developed for a range of uses including monitoring illicit drug usage in communities, screening for viruses and diagnosing conditions such as diabetes. Most studies were nonrandomized, small-scale pilot or lab studies. Of the sanitation-related biosensors found in the literature, 11 gathered population-level data, seven provided real-time continuous data and 14 were noted to be more cost-effective than traditional surveillance methods. The most commonly discussed strength of these technologies was their ability to conduct rapid, on-site analysis. The findings demonstrate the potential of this emerging technology and the concept of *Smart Sanitation* to enhance health monitoring at the individual level (for diagnostics) as well as at the community level (for disease surveillance).

## 1. Introduction

Global health is the area of research and practice that prioritizes improving health and achieving health equity for people worldwide [1]. It emphasizes transnational health issues and embodies the idea that any one country cannot isolate itself from the public health challenges facing other nations [1]. With increased globalization, infectious diseases of today’s world spread and affect larger populations more rapidly than they ever have before. Global threats require global solutions that are unified; therefore, nearly 70 national governments have partnered with international organizations and nongovernmental stakeholders to ‘prevent, detect and respond’ to disease threats [2]. This initiative, the Global Health Security Agenda (GHSA), acknowledges that outbreaks do not respect borders and that a pathogen can travel across continents in a matter of hours [2]. GHSA is multisectoral and multilateral; in addition to governments, GHSA membership includes the Global Health Security Agenda Consortium, a collective of nongovernmental organizations, including RTI International (formerly Research Triangle Institute) and Water Aid, as well as academic institutions [2]. Since its inception in 2014, the GHSA has informed the response to epidemics like Novel Coronavirus (COVID-19), Zika virus, Avian Influenza and the Ebola Virus [2]. In 2019, the United States released its own Global Health Security Strategy to affirm its commitment to the GHSA and outlined its plans for responding to infectious disease threats including Public Health Emergencies of International Concern (PHEICs), as declared by the World Health Organization (WHO) [3].

In addition to being international, GHSA initiatives are multi-sectoral, involving improvements across clinical, agricultural and technological spheres. Similarly, the WHO calls for innovative ways to connect communities, non-governmental organizations and private sector companies to improve health. WHO outlines priorities for strengthening health systems around the globe by expanding effective health services and ensuring equitable access to medical products and technologies [4]. The water, sanitation and hygiene (WASH) sector is a key area to target such improvements as it has the potential to prevent at least 9.1% of the global disease burden and 6.3% of all deaths [5]. Adequate WASH infrastructure is essential for providing quality healthcare, improving infection control, reducing the spread of resistant pathogens, promoting social and economic development and maintaining personal dignity. Thus, the United Nations (UN) 6th Sustainable Development Goal (SDG) aims to “achieve access to adequate and equitable sanitation and hygiene for all and end open defecation, paying special attention to the needs of women and girls and those in vulnerable situations” by 2030 [6]. Adequate sanitation must be safely managed throughout the sanitation chain and improved sanitation services are those that separate excreta from human contact, including improved pit latrines, pour-flush toilets, flushing toilets, sewage systems and septic systems [7].

These services are regularly monitored to ensure that infrastructure is properly functioning and monitoring health outcomes, such as infectious and chronic diseases, could be integrated into this process. Improved sanitation services can be leveraged for public health surveillance using emerging technologies that examine the characteristics of waste to collect real-time data on human health and behaviors [8]. The concept of *Smart Sanitation* could be a useful approach in making health monitoring more efficient, comprehensive and less resource intensive. The Toilet Board Coalition defines *Smart Sanitation* as a way to build resilience in cities, communities and sectors by utilizing Fourth Industrial Revolution technologies to improve the collection and monitoring of wastewater for both individualized and aggregate-level preventative health surveillance [8].

In the past decade, wastewater-based epidemiology has emerged as an innovative approach to survey and evaluate population-level exposure to chemical and biological compounds by analyzing biomarkers found in sewage [9,10]. Biomarkers are useful for characterizing exposures specific to human consumption as they can target metabolites and excretion products that are not associated with environmental sources [11]. Currently, biomarkers are being developed and validated to quantify community consumption of pharmaceuticals, illicit drugs and pesticides, as well as to detect specific diseases [11]. Compared with traditional survey methods, wastewater-based epidemiology is cost-effective, rapid and does not rely on self-reported data [11]. Still, biomarker data often require off-site preparation and analysis by trained laboratory personnel, which are limitations that could potentially be addressed with autonomous biosensor technologies.

Biosensors input biological responses that occur as a body’s reaction to an external stressor, such as variation in pH, temperature or enzymatic activity and convert them into a measurable, electrical signal [12]. Today, digital technologies increasingly interact with the biological world and biosensors have become a powerful tool in quantifying the health of human bodies and populations [13]. Related to sanitation, biosensors can enhance population health monitoring and prevent outbreaks through detecting specific biomolecules in urine and wastewater samples [11]. Biosensors can also be used to assess health interventions with little cost to research participants [14]. In addition to wastewater-based epidemiology, biosensors can assess the health of individuals by monitoring behaviors such as toilet usage and health indicators such as urine flow [15]. Biosensors are currently being developed for use in industrial wastewater treatment facilities and drinking water systems for quality control and early detection of system failures [16,17]. The WASH field is a prime area for the development and implementation of biosensor technologies in lieu of traditional methods for sample collection and analysis. This literature review addresses current research surrounding biosensors as a tool for public health surveillance and individual health monitoring which could be applied to reach global health security and health systems strengthening goals. This systematic review will assess how biosensors are being used in sanitation infrastructure to monitor human health.

## 2. Materials and Methods

This study follows the Preferred Reporting Items for Systematic Reviews and Meta-Analyses (PRISMA) statement [18].

### 2.1. Inclusion and Exclusion Criteria

Studies about the use of biosensors in WASH infrastructure were included if they were written in English and were written between the year 2000 and the time of our systematic review. Both gray literature and peer-reviewed literature were included. Papers were excluded if the use of biosensors was primarily implemented for environmental monitoring or other outcomes not directly related to human health. The full inclusion and exclusion criteria are listed in Table 1.

### 2.2. Data Sources, Search Terms and Screening for Inclusion

A systematic literature search was conducted from January to April 2020 to identify all relevant research and trials that met the inclusion criteria. The literature search was conducted in electronic databases (Embase, CDC Stacks, Nexis Uni, PubMed and Global Health) to gather peer-reviewed articles and gray literature. Searches used the following keywords—(i) terms related to WASH (“sanitation,” “toilet,” “sewage,” “wastewater-based epidemiology”); (ii) “global health”; and (iii) terms related to biosensors (“health sensor,” “biosensor,” “biomarker,” “biological sensor”).

Each database was assigned to a reviewer. Abstracts and titles of retrieved literature were screened for relevance and those that were clearly ineligible were excluded (e.g., articles written in a language other than English or published before the year 2000). Retrieved articles and reports that were not excluded after the initial screening were reviewed using a checklist comprising all exclusion and exclusion criteria presented in Table 1. Articles that passed this second level of screening were included for data extraction and all excluded articles were recorded in an Excel spreadsheet.

### 2.3. Information Extraction and Quality Assessment

After study selection, study characteristics including study design, country where the study was conducted, whether data collected was at the individual or community level, type of technology applied, study setting (toilet or latrine, community sewage system, treatment facility, etc.), purpose of sensor (to detect a virus, hormone, pharmaceutical substance, etc.) and specific strengths and weaknesses identified by paper authors were recorded in Excel. A second reviewer verified study selection and data extraction. Disagreements were resolved by a third reviewer. Reviewers coded the data and generated themes following the reflexive thematic analysis process [19]. Deductive and inductive codes were created and applied to the included papers. Codes such as “rapid,” “ease of use,” and “works remotely” were applied to the text only when directly mentioned by the study authors rather than as an interpretation of the data by the research team. Themes were derived from the explicit content of the data following a semantic approach [19]. No meta-analysis was conducted because of the low number of studies eligible for inclusion and the heterogeneity of study aims and measures.

To assess the strength of reporting in selected papers, reviewers evaluated adherence to the Strengthening the Reporting of Observational Studies in Epidemiology (STROBE) statement for observational studies [20]. If the STROBE guidelines were not applicable to the study design, reviewers assessed the limitations and potential sources of bias both reported by study authors and identified by the research team. Risk of bias was not used as exclusion criteria but was reported in the results and discussion sections.

## 3. Results

**Figure 1:**
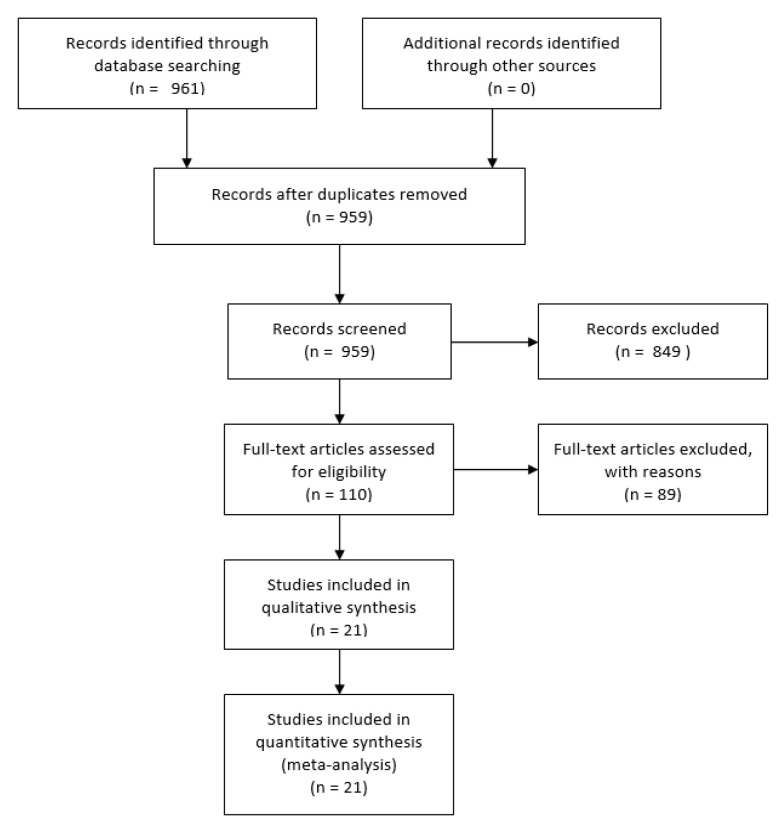
Study Selection.

This systematic literature review retrieved a total of 961 articles. After duplicates were removed, 959 papers were screened for eligibility. After screening the abstract for relevance, 849 papers were excluded and an additional 89 articles were excluded after a full-text review, as they did not fit the inclusion criteria outlined in Table 1. A total of 21 articles (18 peer-reviewed and 3 articles published in gray literature) were included in analysis (Figure 1, Table A1).

### 3.1. Study Characteristics

Table 2, Table 3 and Appendix Table A1 summarize the characteristics of the included papers. There was heterogeneity in the country of study, as there were 15 countries and one multi-country study represented among the 21 papers. The majority of these articles described biosensors in laboratory experiments and the remaining were pilot studies and editorials. Biosensors were used to gather individual- and community-level data. Most data were collected in toilets or wastewater systems, but others included testing patient samples of excreta in a clinical setting and even agriculture, where estrogen levels were measured in urine which could both assess human health and the suitability of urine as fertilizer [21]. Of the biosensors used in toilets, studies also included “smart toilets,” self-contained and autonomous technologies that analyze the user’s urine and feces for personalized health monitoring.

Biosensors in these studies were developed for a variety of uses in sanitation infrastructure which ranged from gathering data about specific health outcomes, such as quantifying drug consumption or screening for infectious diseases, to monitoring individual health-related behaviors, such as tracking latrine/toilet usage. Many biosensor and technology types were represented, including DNA biosensors that could detect both cancer markers and pathogens [30], disposable carbon electrode sensors to detect pathogenic bacteria [39] and smart toilets that quantify CO_2_ as a proxy measure for microbiome health [22].

### 3.2. Effectiveness of Biosensors as Described in Included Papers

Among the 21 studies, five of the sensors were described as ready for use in toilets or wastewater systems while nine required further design improvements or trials. The large proportion of biosensor technologies in the prototype stage demonstrates that their use in sanitation is a relatively new and growing field.

The majority of papers mentioned that the biosensors were cost-effective and produced results more quickly compared to the current standard method for detection. Many of the biosensors detected the target substance on site and in real time rather than traditional surveillance methods that often require intermittent manual sample collection and off-site laboratory analysis. As Yang and colleagues described in a paper about using biosensors to monitor community health in sewage systems:

“Compared to conventional analytical tools, biosensors can provide rapid response times, ultrasensitive detection of biomolecules and the potential to be miniaturized for portable assays requiring minimal sample processing.”

[11]

Biosensors have the ability to continuously collect data, often remotely/hands-off, reducing the resources needed to collect information and improving the ability to detect outcomes early or serve as early warning systems. The majority of sensors in this study were portable, allowing for samples to be taken in a variety of locations. Passive data collection using biosensors is often less invasive than other methods, such as blood tests or colonoscopies [23,24] and is more reliable than self-reported data regarding health behaviors [25]. When discussing the benefits of smart toilets specifically, Park and colleagues explained that:

“The intersection of continuous health monitoring and the valuable clinical information obtained from analyzing human excreta lies in the smart toilet. This toilet system is expected to have a major impact on health monitoring research, as the toilet enables longitudinal monitoring of human health with minimal interference of human behavior. It enables patients to reliably obtain data for their own health as well as enabling investigators to conduct large clinical trials.”

[26]

Many papers stated limitations of the use of biosensors, either from their performance in the current study or in future applications. Lab experiments conducted in ideal conditions, especially those that tested artificially prepared samples, would require further testing of biosensors for direct use in wastewater and sewage systems. While papers often cited that maintenance needs of biosensors were less intensive than current methods, smart toilets and other sensors often require regular cleaning [31,41]. One paper specifically states that, “Most of the bacterial biosensors that are currently available are large, require electronics or are too complicated to operate in [developing] countries” [25].

Specifically concerning sensors in toilets, the effects of environmental variables, including whether the user was standing or sitting and the presence of irregular airflow, could affect the biosensor results [25,26,31,38]. One paper explicitly mentioned that while user acceptability of biosensors in toilets “was within an acceptable range,” participants expressed concerns about “privacy protection and data security” as well as the use of cameras identifying toilet users by “analprint recognition” [26]. Authors also raised concerns about the need to validate the lifetime of the biosensor in hot operating environments [24].

### 3.3. Bias and Study Quality

Most of these studies were small-scale, non-randomized pilot or lab studies. Only four [22,24,25,38] papers were eligible to be assessed for reporting quality with the STROBE guidelines for observational studies and all four met at least 17 of the 22 STROBE reporting criteria. While 19 out of the 21 papers were published in peer-reviewed literature, many of these biosensors are in a pre-experimental phase. The lack of randomization, representative samples and adequate controls present among papers in this literature review warrants caution when drawing any generalizable conclusions. Due to these limitations and the nascent nature of this field, further product implementation and research are needed to determine whether biosensors offer improved detection of outcomes of interest compared to the current standard of care.

## 4. Discussion

### 4.1. Strengths and Weaknesses of Biosensors in Sanitation Infrastructure

This literature review shows that there is interest in continued prototyping and piloting of biosensors in sanitation infrastructure. These emerging technologies have the potential to gather preventative and behavioral health data about individuals and populations that could save time and money compared to traditional methods. From as early as 2014, mainstream publications and companies have promoted the concept of *Smart Sanitation* by predicting and provocating that sewage systems will become ‘smart’ and could include ‘lab-on-chip biosensors’ which will permit continuous data collection and real-time surveillance for viral outbreaks at the aggregate level [14]. As one of the papers in our literature review stated:

“Infectious diseases require rapid or even real-time detection to assess whether there is a need for the containment of the disease carriers to certain areas and prevent the development of an epidemic. To this end, there is a need to develop novel analytical tools that are able to accurately and rapidly monitor low levels of biomarkers/pathogens with minimal sample processing by unskilled personnel at the site of sample collection.”

[11]

Overall, our findings demonstrate that biosensors are being developed for use in the sanitation sector and preliminary results indicate that these biosensors can collect useful health-related data. Additionally, biosensors could be used to supplement standard tests that are already in place, such as colonoscopies, surveys or blood work testing to monitor individual and population health more holistically.

However, many papers noted that despite the promising advantages that biosensor technology can bring to health systems, this is a nascent field; therefore, it requires continued rigorous research and product development to reach its potential. Additionally, the lifespan of these various biosensors from both a hardware and surveillance perspective is largely unknown since the majority of the studies were lab-based or pilot studies [24]. These systems may be less accurate when applied to complex matrices such as sewage due to the variable mix of multiple contaminants, pathogens and physical trash (such as used diapers or menstrual health products) that perhaps were beyond the scope of many of the lab-based tests [11]. Some experiments that did test real wastewater, sewage or human specimen samples in the lab tried to account for temperature and other conditions that would be encountered in the field, but these technologies would still require pilot testing to ensure they would be reliable on site.

Additionally, there were some limitations that were only mentioned in a few papers (such as data privacy and impracticalities for low-resource settings [25,26]), while some limitations were not mentioned explicitly but are feasible (such as the potentially high costs related to biosensor technology or installation [26,27]). One paper also reported that “it is uncertain whether the algorithm [that was used with the biosensor to detect ‘likely defecation events’] detects child feces disposal, child latrine training and menstrual [health] management events as ‘likely defecation events’,” which is also a notable limitation [25]. The majority of the actual limitations reported for biosensors embedded in sanitation infrastructure are technological rather than methodological barriers; therefore, they have the potential to be improved through iterative changes in the various devices’ product designs and fine-tuning their technological prowess.

Despite the current limitations of biosensors, the long-term benefits outweigh the barriers. Public health stakeholders and WASH practitioners should not be deterred from adopting biosensors into sanitation products and infrastructure and should seek to further build the evidence base to determine the true global health potential that this *Smart Sanitation* technology could have.

### 4.2. Population- and Individual-Level Health Considerations of Smart Sanitation

Transforming sanitation infrastructure into a health diagnostic tool has implications both on the population and individual levels. At the population level, decision makers and public health practitioners could use biosensors that have been integrated into public sanitation infrastructure to mitigate and prevent infectious disease outbreaks [11]. On the individual level, there is also a demand for personal health monitoring. Similar to the biometric sensors integrated into consumer products such as the Apple Watch [42] or Fitbit [43], there appears to be a growing demand for individuals to use *Smart Sanitation* products to inform their lifestyle decisions and personal health investments. There are already at-home diagnostic methods available, such as Cologard’s colon cancer screening kit where individuals can mail stool samples to be tested [44], which have potential to be improved and streamlined with the use of biosensors.

There has been an influx of *Smart Sanitation* private sector products on the market, such as TOTO’s Flow Sky Toilet in 2018, a toilet that is equipped with a biosensor to capture the flow rate of the user’s urine as an integrated urological medical examination [15], and Pampers’ ‘smart diaper’ in 2019, which tracks an infant’s sleep and urine patterns which is oftentimes a recommended practice from pediatricians [45]. These technologies represent the burgeoning market and demand from consumers for *Smart Sanitation*-related products for monitoring their individual health.

While there are a myriad of innovative opportunities for further research in the field, it is imperative that these products account for data privacy. Capturing data about individual-level health data, as opposed to information gathering about population-level health, must often adhere to regulatory standards such as the Health Insurance Portability and Accountability Act (HIPAA) Privacy Rule [46] in the United States of America and the General Data Protection Regulation (GDPR) [47] in the European Union. *Smart Sanitation* consumer products should be developed in such a way that is in line with these standards to safeguard individual-level health data while also fostering consumer trust as a result of transparent data protections in these products.

### 4.3. The Covid-19 Era: Furthering the Case for Increased Smart Sanitation Research

The need for embracing and further researching the implications of *Smart Sanitation* has been underscored by the widespread societal implications of the Novel Coronavirus (COVID-19). Recent studies have also shown SARs- CoV-2, the virus causing COVID-19, is shed in feces which means that fecal-oral contamination could be another route of transmission [48]. Biobot, a wastewater epidemiology startup, has conducted opioid and COVID-19 wastewater analytics and monitoring within the United States to create data-driven maps for community-level decision makers to see as the disease spreads in real time [49]. Researchers in Spain are now collecting samples approximately twice a week from over 250 wastewater treatment facilities for improved COVID-19 surveillance through sewage [50]. This would be an ideal place to implement biosensors in sewage systems, which would allow for this data collection to occur more quickly with less potential for exposure for field workers and lab technicians.

However, due to the nascent nature of this sector, the preventive health benefits of the *Smart Sanitation Economy* will likely not be realized quickly enough to meaningful impact the COVID-19 pandemic. Beyond current pathogenic testing, next generation *Smart Sanitation* biosensors could be made to detect a wide array of biomarkers to mitigate and monitor future epidemics.

### 4.4. Limitations of this Review

This review has a number of limitations. First, identifying findings that explicitly explained product/device failures or misuses in this sector was difficult. While this could be attributable to publication bias, it could also be because the field is so new that few reports of product trials exist at all or they are not publicly available. Second, the research team exclusively searched for gray literature in the Nexus Uni database, which may have missed internal product reports, news articles and other relevant information. Third, academic literature on this subject tended to focus on the mechanics of the technologies rather than the practicalities of the device in practice (such as cost, maintenance and usability), which is likely because these technologies are largely in the pre-implementation phase.

## 5. Conclusions

The findings of this systematic review demonstrate the public health potential of biosensors as an emerging technology and how *Smart Sanitation* can be used to enhance health monitoring at the individual level as well as at the community level. Most studies were nonrandomized, small-scale pilot or lab studies and revealed that few biosensors have been used in toilet or human sewage settings outside of lab tests. Many of the biosensors provided real-time continuous data and were reported to be more cost-effective in terms both time and money when compared to traditional surveillance methods. The most commonly discussed strength of these technologies was their ability to conduct rapid, on-site analysis. The use of biosensors in sanitation is a nascent field that has the potential to promote global health security and strengthen health systems. In order to effectively develop and implement these technologies for public health surveillance and individual health monitoring at a large scale, more robust research is needed to evaluate and improve existing biosensors technologies to not only ensure the efficacy of their use but also their compliance with global data privacy and human study protocols. Additional research in this field could also identify gaps where next generation versions of the biosensor technologies can emerge.

## Figures and Tables

**Table 1:** Inclusion and Exclusion Criteria.

Category	Included	Excluded
Publication Language	English	
Study types and designs	Systematic reviews, random controlled trials, product pilots/trials (non-random), program reports, news articles describing the use of biosensors by organizations	
Publication Type	Peer-reviewed articles and gray literature (including program reports and reputable news articles about product pilots)	Textbooks, product manufacturer documents such as schematics, patents
Intervention type	WASH interventions involving biosensors aimed at improving human health either at a public health (e.g., biosensors used in wastewater treatment plants to evaluate effectiveness of filtration systems) or personal health (e.g., biosensors in toilets in participant’s homes to alert the presence of a certain bacteria) level	Interventions that do not utilize a biosensor, do not relate to human waste and sanitation (e.g., environmental/ecological monitoring as the sole use of biosensors, sensors used in industrial wastewater or sensors applied to drinking water) or were not directly related to a human health outcome
Publication dates	2000 to the present	Literature dated 1999 and prior

**Table 2:** Study Characteristics.

Codes	Frequency	Reference Number
**Intended Setting for Use of Biosensor**		
Toilet	8	[22,23,24,25,26,27,28,29]
Wastewater	8	[11,30,31,32,33,34,35,36,37]
Bathroom	1	[38]
Patient Stool/Urine Sample in clinical setting	3	[39,40]
Agriculture	1	[21]
**Purpose of Sensor/Health Outcome Measured**		
(Biosensors that measured multiple health outcomes are repeated)		
Diagnosis of a health condition (e.g., diabetes)	4	[23,26,39,40]
Measure temperature	1	[24]
Measure gut health (CO_2_ or microorganisms)	2	[22,28]
Measure blood sugar	1	[27]
Detect presence or quantify of medications or illicit drugs	4	[31,32,36]
Detect Biomarkers of Disease	4	[11,30,39,40]
Determine latrine/toilet usage	3	[25,29,38]
Detect chemicals/heavy metals	3	[34,35,37]
Detect Estrogen	1	[21]
Estimate population size by detecting human population biomarkers	1	[33]
**Study Type**		
Lab/experiment	16	[21,23,26,27,28,29,30,31,32,33,34,35,36,37,40]
Pilot study	4	[11,22,24,25,38]
Editorial	1	[11]
**Population of Interest**		
Individual	10	[22,23,24,27,28,29,31,38,39,40]
Community	11	[11,21,25,26,30,32,33,34,35,36,37]
**Samples Tested**		
Artificially prepared sample (e.g., chemical added to purified water)	5	[9,14,15,18,21]
Unmodified sample collected from the field or test subject (e.g., wastewater or sewage samples)	13	[1,2,3,4,6,7,10,11,13,16,17,19,20]
No samples taken (e.g., motion detection for latrine use)	3	[5,8,12]
**Article Type**		
Peer-reviewed	18	[21,22,24,25,26,27,29,30,31,32,33,34,35,36,37,38,39,40]
Gray Literature	3	[11,23,28]
**Author’s Stated Next Steps**		
Improve sensor and/or resolve errors	9	[1,2,4,5,8,10,16,18,20]
Clinical Trials	2	[9,13]
Ready for use in toilets or wastewater systems	6	[7,11,14,15,17,21]
Unspecified/no next steps stated by the author	4	[3,6,12,19]

**Table 3:** Themes of interest.

Codes	Frequency	Reference Number
**Biosensor Characteristics**		
Portable	11	[11,22,23,24,25,30,33,34,35,38,40]
Remote/Hands-off	7	[11,24,25,26,28,36,38]
On-site analysis (no separate lab test needed)	12	[11,22,23,24,26,27,28,29,34,35,36,40]
Continuously gathering data/longitudinal data collection	7	[11,24,25,27,28,31,38]
Easy to use without extensive technical knowledge, time or money involved	9	[21,22,24,27,30,32,37,38,40]
**Comparison to Current Methods**		
More Rapid than conventional data collection methods	8	[11,23,26,32,33,34,35,39]
Less Rapid than conventional data collection methods	None mentioned	n/a
Higher user acceptability/less invasive	7	[22,23,24,25,26,27,38]
Less user acceptability due to obstruction of their daily behaviors, feeling discomfort from being surveyed, etc.	1	[26]
Low Cost	14	[11,21,23,24,25,30,31,32,33,34,35,37,38,39]
High Cost	None mentioned	n/a

## Appendix

**Table A1:** Study Characteristics of Included Papers.

Author, Publication Year, Country [ref]	Technology Used	Setting of Use in Study
Yang et al., 2015, Europe (multiple) [11]	Multiple (review paper)	Wastewater
Jaatinen et al., 2016, Finland [21]	Whole cell sensor	Urine samples
Lucarelli et al., 2002, Italy [30]	Carbon electrode sensor	Wastewater
Webster et al., 2014, United States [39]	Carbon electrode sensor	Urine samples
Hirayama et al., 2010, Japan [22]	Infrared	Toilet
Soh, 2005, Singapore [23]	Electric biosensor	Toilet
Kawanami et al., 2012, Japan [24]	Thermocouple sensor	Toilet
Delea et al., 2017, Bangladesh [25]	Motion sensor	Toilet
Park et al., 2020, United States [26]	Smart toilet, DNA biosensor	Toilet
Masemola et al., 2020, South Africa [31]	Electric biosensor	Water
Chapron et al., 2019, Canada [38]	Infrared	Toilet
Ghosh et al., 2020, India [27]	Smart toilet, Transducer	Toilet
Porecha, 2015, United States [28]	Smart toilet	Toilet
Nakagawa et al., 2018, Japan [29]	Electrocardiogram	Toilet
Mao et al., 2019, China [32]	Colorimetric biosensor	Wastewater
Yang et al., 2015, United Kingdom [33]	DNA biosensor	Wastewater
Timur et al., 2004, Turkey [34]	Whole cell sensor	Wastewater
Gao et al., 2017, China [35]	Whole cell sensor	Wastewater
Pham et al., 2015, Germany [36]	Whole cell sensor	Wastewater
Gagnon & Lajeunesse, 2008, Portugal [37]	Whole cell sensor	Wastewater
Bunyakul et al., 2015, Thailand [40]	Microfluidic immunosensor	Stool samples
